# Clinical Management of Ovarian Function Suppression in Premenopausal Women With Breast Cancer: A Survey of Members of ASCO

**DOI:** 10.1200/OP-24-00502

**Published:** 2024-11-12

**Authors:** Catherine M. Kelly, Kathleen E. Bennett, Caitriona Cahir, Andrea Eisen, Lajos Pusztai

**Affiliations:** ^1^ Department of Medical Oncology, Mater Private Hospital, Dublin, Ireland; ^2^ School of Population Health, RCSI University of Medicine and Health Sciences, Dublin, Ireland; ^3^ Odette Cancer Center, Sunnybrook Health Sciences Centre, Toronto, Canada; ^4^ Juravinski Cancer Centre, McMaster University, Hamilton, Ontario, Canada; ^5^ Center for Breast Cancer, Smilow Cancer Hospital, Yale, New Haven, CT

## Abstract

**PURPOSE:**

Ovarian function suppression (OFS) with gonadotropin-releasing hormone agonists (GnRHas) is a standard of care for premenopausal patients with high-risk stage II/III hormone receptor–positive breast cancer (BC). Practical guidance on the optimal choice of GnRHa, timing, schedule, and monitoring is limited. Our aim was to determine how oncologists use OFS in routine care.

**METHODS:**

We designed a questionnaire to determine the choice of GnRHa, schedule, duration, initiation, use of bone modifiers, and monitoring of estradiol (E2). The questionnaire was sent to oncologists treating BC, in practice for >1 year and participating in the ASCO Research Survey Pool (RSP). It was also forwarded by investigators to oncologists meeting these criteria. The survey was open between November 14, 2023, and January 5, 2024.

**RESULTS:**

Of 996 oncologists participating in the ASCO RSP, 178 (18%) completed the survey. An additional 56 oncologists contacted by investigators responded. Respondents were from the United States (57%), Asia (15%), and Europe (14%). Goserelin (54%) and leuprolide (39%) were the most frequently used GnRHas and were administered once every month by 46%. Approaches to starting GnRHas were varied. Most continued them for the duration of aromatase inhibitor therapy (57%). Estradiol monitoring was performed regularly, sometimes, or never by 43%, 27%, and 27%, respectively. The E2 assays used were standard (65%), ultrasensitive (16%), and unknown (14%). Interpreting E2 assay results were considered difficult by 55%; however, 62% of oncologists changed treatment on the basis of them. A total of 92% of respondents would like ASCO guidance on the practical use of OFS.

**CONCLUSION:**

Considerable practice variation exists for similar clinical scenarios in OFS administration. Respondents would welcome ASCO guidance on all aspects of OFS.

## INTRODUCTION

Globally, women younger than 50 years comprise 30.9% of all breast cancer (BC) cases and hormone receptor–positive disease is the most common subtype.^1^ Meta-analyses showing a 15% reduction in BC mortality with 5 years of an aromatase inhibitor (AI) compared with 5 years of tamoxifen led to AIs becoming the preferred option for postmenopausal women.^2^ AIs are contraindicated in premenopausal women as inhibition of aromatase causes an initial decline in estradiol (E2), which stimulates release of follicle-stimulating hormone (FSH) and luteinizing hormone (LH) from the anterior pituitary, resulting in ovarian E2 production. In premenopausal patients with chemotherapy-induced amenorrhoea, AIs can stimulate E2 production in those who regain ovarian function, which can happen even after several years from AI initiation.^3-5^

**CONTEXT**

**Key Objective**
Does significant practice variation exist in the use of gonadotropin-releasing hormone analogs for ovarian function suppression in premenopausal breast cancer?
**Knowledge Generated**
In a survey of ASCO members, there was significant practice variation in all aspects of gonadotropin-releasing hormone agonist (GnRHa) use. Variations exist in the agents used, duration and schedules, starting approaches, frequency of estradiol testing, and types of assays used.
**Relevance**
Medical oncologists require practical guidance on the optimal choice of GnRHa, timing and schedule, duration of use, and monitoring and interpretation of estradiol levels.

Premenopausal women can receive AIs in conjunction with ovarian ablation (oophorectomy or ovarian irradiation) or ovarian function suppression (OFS) with gonadotropin-releasing hormone receptor agonists (GnRHas). Continuous administration of a GnRHa desensitizes and downregulates GnRH receptors and inhibits production of FSH/LH causing OFS and a decline in serum E2 levels.^6^ Unlike ovarian ablation, administration of a GnRHa causes reversible OFS and is preferred in younger women and those planning future pregnancies.

The benefit of OFS/ablation has been observed in individual and joint analyses of The Suppression of Ovarian Function Trial (SOFT) and the Tamoxifen and Exemestane Trial (TEXT) clinical trials.^7-12^ These trials have shown that tamoxifen alone is associated with an 8-year freedom from distant recurrence of more than 97% in women with lower-risk cancers (ie, not requiring chemotherapy, ≤2 cm, and low histologic grade). By contrast, gains of 10%-15% absolute improvement in 8-year freedom from distant recurrence were observed in women with high clinicopathologic risk with exemestane plus OFS versus tamoxifen plus OFS or tamoxifen alone.^11^ A meta-analysis of 15,000 women from 25 randomized trials examining the addition of OFS/ablation showed an improvement in 15-year risk of BC recurrence of 12.1% and mortality of 8.0% and all-cause mortality of 7.2% for the addition of OFS/ablation in premenopausal women.^13^ Similar improvements in survival were observed in a Cochrane review that examined OFS versus no OFS in 11,500 premenopausal women with hormone receptor–positive BC over 15 studies.^14^

Despite the establishment of OFS as a part of standard-of-care adjuvant therapy for premenopausal women with hormone receptor–positive BC at high risk of recurrence, guidance on the practical administration and monitoring of it is limited. Comparative efficacy data for the various available GnRHas are also limited. Most clinical trials have used a once every month GnRHa schedule (referred to as monthly), and this is the preferred option of the ASCO Clinical Practice Guideline Update on Ovarian Suppression and the European School of Oncology (ESO)-European Society of Medical Oncology (ESMO) fifth international consensus guidelines for BC in young women (BCY5).^15,16^ However, 3 and 6 monthly (ie given once every 3 months or once every 6 months) formulations of these drugs are available and represent a more convenient option for patients.^17-23^ Clinical trials did not routinely monitor E2, FSH, and LH levels during GnRHa therapy. Multiple studies have reported inadequate E2 suppression during GnRHa therapy, leading the investigators to advocate for routine hormone profile (ie, E2, FSH, LH) monitoring.^4,5,24-27^ The ASCO and ESO-ESMO BCY5 guidelines recommend hormone profile testing when inadequate ovarian suppression is suspected on the basis of clinical symptoms (eg, resumption of menses or cyclical fluctuations in climacteric symptoms), but routine testing is not endorsed.^15,16^ There are technical limitations with all currently available E2 assays, and there is no universally accepted E2 level for inadequate ovarian suppression.^28^ The aim of this study was to improve understanding of current practice patterns around GnRHa use, assess practice variation, and identify if practical guidance was needed.

## METHODS

We conducted a cross-sectional survey. Participants were identified using the ASCO Research Survey Pool (RSP) available through ASCO's Center for Research and Analytics (CENTRA). The ASCO RSP includes US and international ASCO members who volunteer to receive surveys. Our survey was distributed to RSP participants who were practicing oncologists, treating BC, and in practice for >1 year. The online questionnaire was distributed via e-mail. Investigators were permitted to forward the questionnaire link to colleagues who met the inclusion criteria. The analyses were conducted for the entire cohort (Data Supplement, online only) and separately for the ASCO RSP respondents and those responding to the link sent by investigators (Data Supplement).

### Survey

We designed a questionnaire with 26 multiple choice questions to elicit how physicians make decisions and administer GnRHas in common clinical scenarios. The survey opened on November 14, 2023, and closed on January 5, 2024. The questionnaire comprised six sections:

1. Respondent demographics
2. What GnRHa do you use? What schedule do you use? Does age influence the dose or schedule? Is schedule influenced by BMI, receipt of chemotherapy, finance, tolerance, no factors, and other—specify? What is your intended duration of use?
3. When do you start a GnRHa relative to chemotherapy or to other endocrine therapy when chemotherapy is not given?
4. Do you use bone-modifying agents during OFS? Which agent(s) do you use? How long do you use them?
5. Do you check a baseline hormone profile? What influences checking baseline hormone levels? What do you check? Do you check during OFS, and how often? How long do you continue checking? What type of E2 assay do you use? Do you change treatment on the basis of E2 result? Do you find interpreting E2 in this setting challenging?
6. Would you like ASCO guidance on testing methods, interpretation, and scheduling of OFS and AI?

Complete survey and the answers are included in the Data Supplement.

### Statistical Analysis

To obtain a representative opinion from the ASCO RSP survey pool of 996 individuals with a 95% CI with ± 6% error on any percentage, we needed 211 respondents. Responses to each question were summarized using frequency counts and percentages. Statistical comparisons were made between different groups using the chi-square statistics to test associations between categorical variables. Nominal *P* values are reported. All statistical analyses were performed using SPSS version 29.1.0.1 (171). This study was exempt from human subject research by the Mater Misericordiae University Hospital Institutional Review Board.

## RESULTS

Nine hundred ninety-six questionnaires were disseminated by ASCO's CENTRA, and 178 of these were returned (18% response rate). Twenty-nine (12.4%) questionnaires were excluded as these respondents were not treating BC. An additional 56 questionnaires were returned by the links sent by the investigators, and the denominator for this group is not available.

A total of 205 were included in the analysis presented below.

### Section 1

Respondents were from the United States and Canada (57%), Asia (15.1%), Europe (14%), South America (4%), Africa (2%), and Australia (2%). Most treated breast and other cancers (67%), treated BC only (29%), or did not specify (3%). Most were practicing for >5 years (79%), and 60% of all respondents managed >20 unique patients with BC each month (Table 1).

**TABLE 1.** Characteristics of all Survey Respondents

Respondent Demographics	Frequency, No. (%)
Continent of residence	
North America	117 (57)
Asia	31 (15)
Europe	29 (14)
South America	9 (4)
Africa	5 (2)
Australia	5 (2)
Missing	9 (4)
Which of the following best describes you?	
Male	108 (53)
Female	84 (41)
Nonbinary	0 (0)
Prefer not to say	6 (3)
Self-identify as	0 (0)
Unanswered	7 (3)
How would you describe your practice	
Practice owned by the hospital or health system^a^	155 (76)
Physician-owned practice (ie, independent, office-based)	38 (19)
Other	2 (1)
Unanswered	10 (5)
How many years have you been treating patients with breast cancer	
<5	35 (17)
5-10	48 (23)
11-15	31 (15)
16-20	29 (14)
21-25	22 (11)
>25	32 (16)
Unanswered	8 (4)
What cancer types do you treat?	
Breast cancer only	138 (67)
Breast and other cancers	60 (29)
Did not specify	7 (3)
Approximately how many unique breast cancer patients do you see each month?	
1-5	19 (9)
6-10	24 (12)
11-20	39 (19)
>20	123 (60)

^a^ Including academic medical centers, government-funded institutions, and integrated health care systems.

### Section 2

Most physicians used goserelin (54%) followed by leuprorelin (39%) and triptorelin (5%). The most common schedule was monthly (46%), followed by 3 monthly (22%), but 30% used either schedule depending on the clinical circumstance. Ninety percent indicated that patient age did not influence GnRHa dose, and 80% indicated that age did not influence the schedule of GnRHa administration. Comments indicated that the 3 monthly schedule was mostly used for older patients or when ovarian suppression had been confirmed (Data Supplement). Other factors influencing schedule were reimbursement/financial (30%) and patient preference/tolerance (26%). Some comments indicated concerns about reduced efficacy with 3 monthly GnRHa administration (Data Supplement). Almost 60% uses a GnRHa for the entire duration of the AI or until natural menopause, and a minority administered GnRHa only for 5 years (32%) or 2 years (9%).

### Section 3

**FIG 1.**
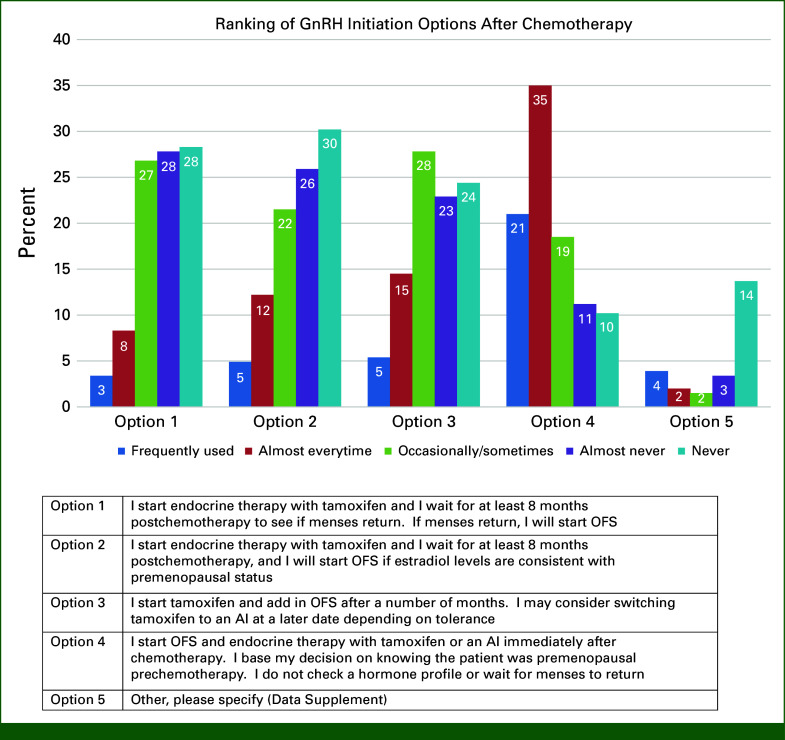
Ranking of GnRHa initiation options after chemotherapy. AI, aromatase inhibitor; GnRH, gonadotropin-releasing hormone; GnRHa, gonadotropin-releasing hormone agonists; OFS, ovarian function suppression.

We provided four approaches to initiating a GnRHa with tamoxifen or AI after chemotherapy. Fifty-six percent almost every time or frequently start the GnRHa with tamoxifen/AI immediately after chemotherapy, if the patient was premenopausal before chemotherapy, and they do not check a hormone profile or wait for menses to return (Fig 1). Comments refer to many other approaches (Data Supplement).

**FIG 2.**
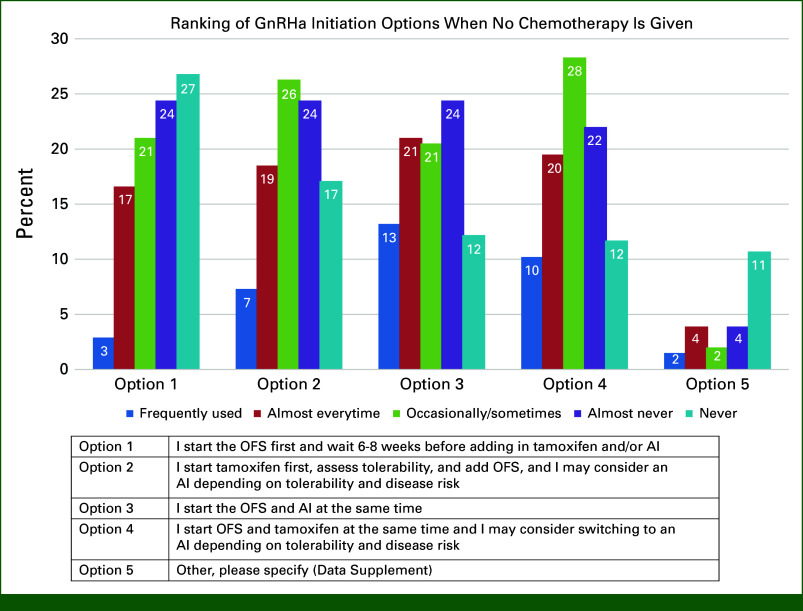
Ranking of GnRHa initiation options when chemotherapy is not given. AI, aromatase inhibitor; GnRHa, gonadotropin-releasing hormone agonist; OFS, ovarian function suppression.

We provided four approaches to starting a GnRHa when chemotherapy is not given (1) start and wait 4-6 weeks before adding tamoxifen or an AI; (2) start tamoxifen, assess tolerability, then add the GnRHa, and consider AI on the basis of tolerance and disease risk, (3) start GnRHa and AI simultaneously; and (4) start GnRHa and tamoxifen simultaneously and consider switch to AI depending on tolerance and disease risk. No option was clearly favored (Fig 2). Twenty-two percent (n = 45) indicated taking a different approach, but only 18 individuals (9%) detailed their approach, and of them, 13 started the GnRHa first and introduced an AI between 2 and 24 weeks later (Data Supplement).

### Section 4

Almost 60% use a bone-modifying agent sometimes (59%), and 30% always (28%). Table S1 (Data Supplement) shows the agents used and frequency. Almost half used these agents for 3 years (46.8%), and 30 percent used them for 5 years (29.3%). Of the 10% of respondents who use another duration, these included 2 or between 3 and 5 years of treatment. Factors influencing treatment duration are tabulated in the Data Supplement.

### Section 5

**FIG 3.**
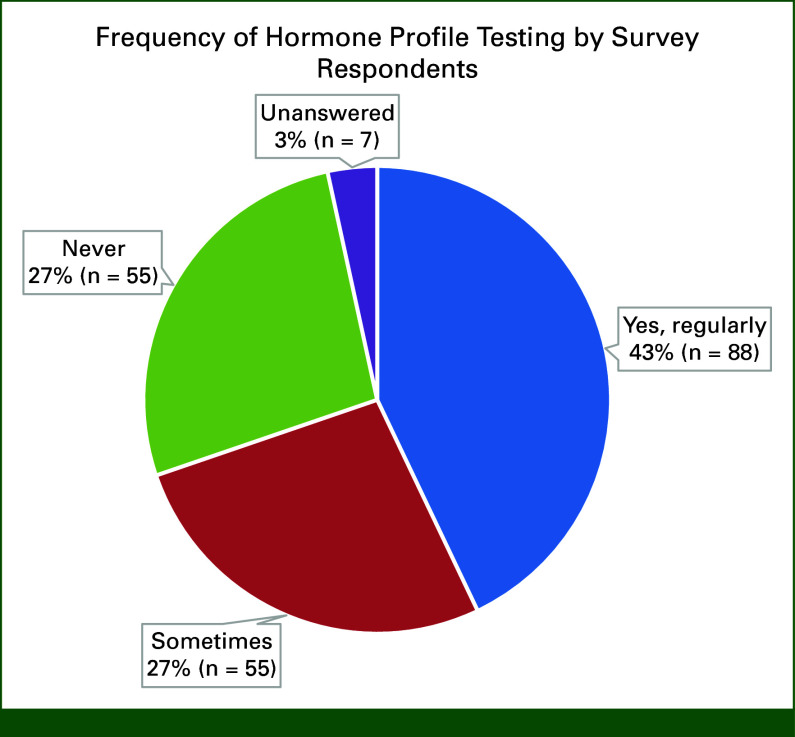
Frequency of hormone profile testing by survey respondents.

**FIG 4.**
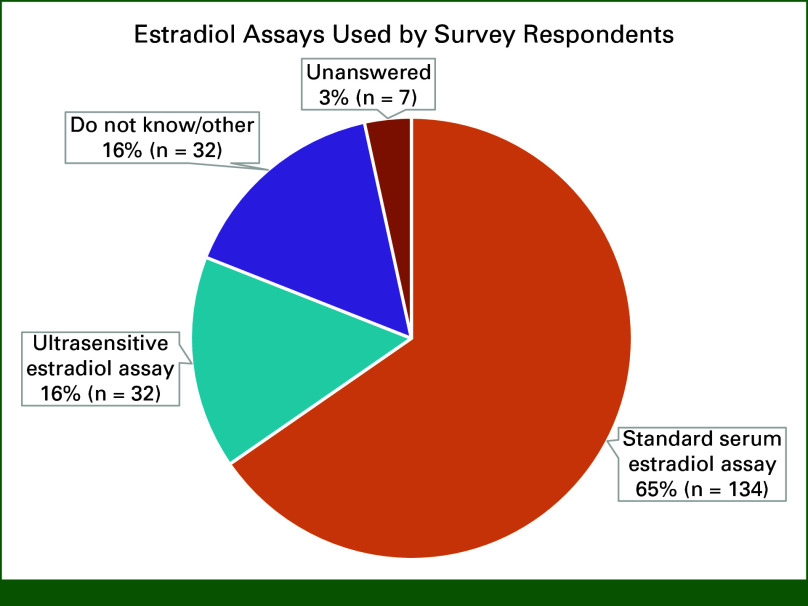
Estradiol assays used by survey respondents.

**FIG 5.**
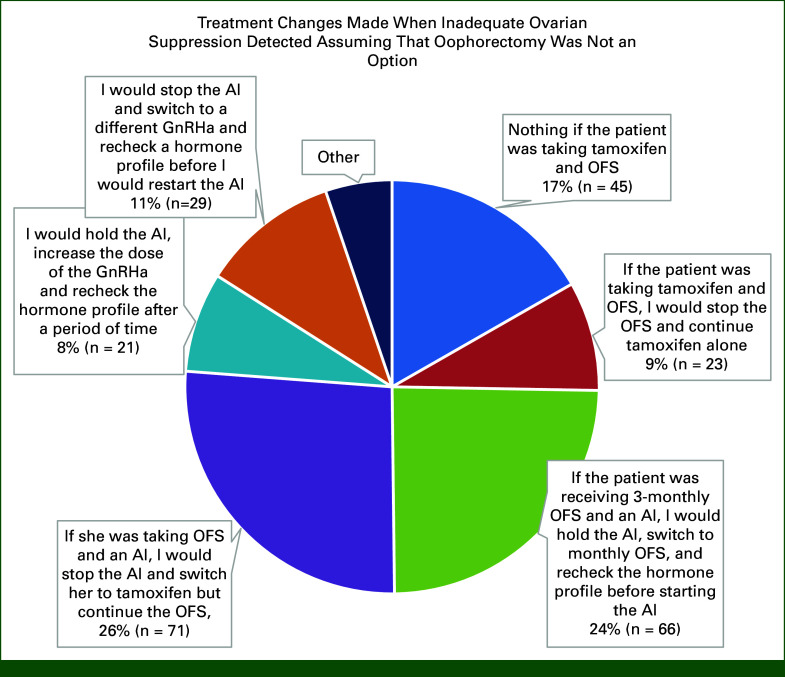
Treatment changes made when inadequate ovarian suppression is detected assuming that oophorectomy is not an option (^a^Survey respondents could select more than one option). AI, aromatase inhibitor; GnRHa, gonadotrophin releasing hormone agonist; OFS, ovarian function suppression.

Ninety-three percent of respondents did not answer if they checked a hormone profile at baseline before GnRHa initiation. It is likely there was a technical issue with this question. By contrast, 43% regularly and 27% sometimes check hormone profiles during GnRHa therapy (Fig 3). Of the 27% checking sometimes, reasons prompting testing were uncertain menopausal status, suspicious symptoms, or return of menses to check if ovarian suppression is adequate, age, administering 3 monthly, financial constraints to continue, and compliance. Test repetition varied, and reasons included “until I am satisfied ovarian suppression has been achieved (n = 33),” “entire duration of planned treatment (n = 25)” and “first 2 years (n = 14)” (Data Supplement). Sixty-five percent surveyed use of standard E2 assays, 16% surveyed use of an ultrasensitive assay, and the remainder did not know which assay is used (Fig 4). Most respondents (62% n = 128) would change treatment if they detected inadequate ovarian suppression, but 32% (n = 65) would not, and 6% did not answer. Figure 5 shows how inadequate ovarian suppression is being managed by those who change treatment. Of the 14 comments, seven favored oophorectomy, and seven would increase the dose or switch schedule from 3 monthly to monthly or shorten monthly to 3 weekly administration. Five of the seven clarified that they would not hold the AI during these changes (Data Supplement). Fifty-five percent found interpreting hormone profiles challenging, and 13% selected “we should not be checking them because they are difficult to interpret and we do not know the clinical significance if any of ovarian escape” (Data Supplement).

### Section 6

The most consistent response in our survey was that 92% of all respondents indicated that they would like guidance from ASCO regarding the practical use of OFS in this setting. Thirty-three percent (n = 67) provided comments describing OFS therapy as “complicated,” “challenging,” “an uncharted area, with numbers of patients increasing,” and that it “needs a focused approach.” Twenty-four respondents stated the specific aspect of OFS they needed guidance on including what drugs to use, schedule, duration, monitoring, and interpretation. Respondents expressed a need for standardization while simultaneously appreciating the complexity of this therapy. There were 30 direct requests for scientifically based, standardized, consistent, and uniform guidance. Comments also acknowledged the challenge of generating guidance given the relative lack of data in this area, the multiple clinical variables, and the need for nuanced approaches. Two respondents expressed concerns regarding guidance (Data Supplement).

Since our survey cohort included both the ASCO RSP respondents and physicians recruited by investigators, we also performed separate analyses by recruitment cohort. The investigator recruited cohort was more likely to treat BC only, be based in Europe, use goserelin more than leuprolide, have the GnRHa schedule influenced by previous chemotherapy and not by reimbursement, use an ultrasensitive assay, and use a GnRHa for 5 years compared with ASCO RSP–recruited participants. Both cohorts found it equally challenging to interpret hormone profiles and wanted guidance (Data Supplement).

## DISCUSSION

Our survey shows very large variations in almost all aspects of GnRHa therapy. Some of these variations may reflect drug availability and the regulatory environment, but others reflect limitations in the literature and lack of consensus guidelines on practical aspects of this therapy. All three GnRHas have been used in adjuvant BC clinical trials, and each is endorsed in at least one guideline developed by ASCO, National Comprehensive Cancer Network (NCCN), or ESMO.^15,16,29^ Interestingly, only 5% of respondents used triptorelin despite its use in several key clinical trials, which may reflect a reimbursement/access issue rather than efficacy concerns.^29^ The NCCN guidelines list goserelin and leuprolide as GnRHa options for OFS.^29^ All three GnRHas suppress E2 levels, but comparative clinical efficacy studies between them in BC are limited.^30^ A systematic review in prostate cancer concluded that all three provide a similar castration effect, but the evidence was insufficient to allow authors to conclude statistical equivalence.^31,32^ Phase II data examining the GnRH antagonist degarelix reported more rapid and sustained ovarian suppression compared with triptorelin, indicating a potential role for these agents, but this agent has not been tested in adjuvant therapy trials in BC.^33^ Comparative efficacy studies are needed.

Almost all adjuvant BC clinical trials have used monthly GnRHa administration, and 46% of respondents selected this as their preferred schedule. This is consistent with the ASCO guideline which not only specifies a preference for monthly administration but also considers 3 monthly a reasonable alternative acknowledging data suggesting similarity in clinical and E2 suppressive effects.^15,21-23,34,35^ Studies have reported younger age as a risk factor for inadequate ovarian suppression, and the ESO-ESMO BCY5 guidelines also state a preference for monthly GnRHa particularly in women younger than 35 years.^5,20,24,25^ However, some studies have not found an association between younger age and higher rate of escape from OFS.^26,27^ Most (80%) respondents indicated that their choice of GnRHa schedule was not influenced by age. The NCCN guidelines list monthly and 3 monthly options and also does not reference age as an influencing factor.^29^ Further studies are needed to determine the significance of age and GnRHa schedule of administration.

Timing of GnRHa initiation is complicated by variations in the definition of premenopausal status in clinical trials.^7,36^ In our survey, when the GnRHa was not started with chemotherapy, most (56%) initiated the GnRHa and AI or tamoxifen immediately after chemotherapy. When chemotherapy was not given, timing of GnRHa therapy was variable. The ASCO and ESO-ESMO BCY5 do not give specific recommendations about when to start the GnRHa in relation to starting endocrine therapy.^15,16^ The NCCN guidelines recommend if no chemotherapy is planned, then the GnRHa should be started alone for at least 1-2 cycles or concurrently with tamoxifen until E2 levels are in the postmenopausal range at which time an AI could be considered.^29^ The TEXT trial required endocrine therapy to start 6-8 weeks after the GnRHa, whereas the SOFT trial allowed exemestane to start any time after GnRHa initiation up to 10 weeks.^7^ Half of respondents never or almost never wait 6-8 weeks after GnRHa inhibition to introduce the AI. The EU summary of product characteristics for triptorelin and leuprorelin in adjuvant BC advises initiating the GnRHa 6-8 weeks before the AI, checking E2/FSH before starting the AI, and performing serial hormone-level testing during AI therapy to avoid AI-induced rebound increase in circulating estrogen, with consequential implications for BC.^37,38^ Our results suggest that education is required to standardize initiation of GnRHa therapy.

We found that the frequency and duration of E2 testing were variable among survey respondents. Most test E2 levels sometime during GnRHa therapy, and 43% test regularly. The ASCO and ESO-ESMO BCY5 guidelines do not recommend routine hormone profile testing unless physiologic changes/symptoms suggest inadequate OFS.^15,16^ By contrast, the NCCN guidelines provide specific clinical scenarios when monitoring is recommended, including age <60 years and amenorrheic for ≤12 months before adjuvant endocrine therapy and if amenorrheic after chemotherapy, taking tamoxifen after switching from tamoxifen to an AI and before the next dose of GnRHa in women younger than 45 years.^29^ As the pivotal adjuvant clinical trials of OFS did not include routine E2 testing to monitor efficacy, it remains unknown if the current symptom-guided approach to E2 testing remains sufficient or if serial testing could optimize therapy. Significantly, 55% of respondents found interpreting hormone profiles a challenge, indicating a need for education.

Although E2 testing is recommended by the ASCO and ESO-ESMO BCY5 guidelines in some situations, neither guideline provide an E2 level that would be indicative of inadequate E2 suppression. The SOFT-EST substudy used an E2 level of <10 pmol/L to indicate ovarian suppression, and this cutoff is being used in the OFSET trial (ClinicalTrials.gov identifier: NCT05879926).^26,39^ Furthermore, E2 thresholds also depend on the assay method. Most respondents (62%) used standard E2 assays that are unlikely to be able to reliably quantify low E2 levels. An Endocrine Society position paper highlighted significant technical issues associated with many commonly used E2 assays.^28^ Although the ESO-ESMO BCY5 guidelines specify a preference for an ultrasensitive E2 assay to monitor therapy, only 16% of respondents use them.^16^ Most hospital laboratories use direct immunoassays optimized to measure E2 concentrations between 40 and 2,000 pg/mL; however, E2 levels of <1 pg/mL (3.67 pmol/L) can be expected in women taking an AI.^28,40^ Oncologists require clear recommendations regarding what E2 assay to use and E2 levels consistent with ovarian suppression. Current guidelines could be improved with input from clinical biochemists and reproductive endocrinologists who can provide more specific evidence-based recommendations.

Most respondents (62%) change treatment on the basis of what they consider inadequate ovarian suppression; however, there were no uniform strategies to manage this clinical situation. This is crucial when an oophorectomy is not an acceptable option for a patient and tamoxifen alone is inadequate because of high risk of recurrence. Treatment changes used by respondents and reported in retrospective studies include continuing the GnRHa but switching from an AI to tamoxifen, shortening the GnRHa administration schedule, increasing GnRHa dose, and changing GnRHa (Fig 5).^24,25^ Surprisingly, 30% of respondents do not change treatment if inadequate ovarian suppression is detected. This may reflect uncertainty regarding the clinical significance of inadequate E2 suppression or difficulties in interpreting hormone profile results. Current guidelines need to provide reasonable management options if inadequate E2 suppression is detected, and studies are needed to determine what strategy provide the best benefit.

Our study has limitations. The response rate from the ASCO RSP was low (18%), and we cannot accurately determine the denominator for the investigator distributed links. However, the ASCO RSP and investigator-recruited participant's results are similar and therefore were presented combined in the article. However, we also provide results separately by recruitment cohorts in the Data Supplement. The relatively low frequency of unanswered questions and the high frequency of voluntary comments indicate that the respondents were a representative sample and invested in this topic.

In comparison with other adjuvant treatment modalities, the administration of OFS is highly variable in clinical practice. Critically, 92% of those surveyed would like guidance on all aspects of ovarian suppression, indicating substantial uncertainty about the optimal way to deliver this potentially lifesaving therapy. Survey responses helped us by highlighting gaps in knowledge around the optimal GnRHa initiation strategies and interpretation of hormone profiles. The survey also drew attention to the variations in current guideline recommendations that contribute to the large variations in clinical practice. Some of the knowledge gaps such as optimal E2 measurements or hormone-level thresholds that define premenopausal status could be clarified through clearer guideline language, and other gaps including optimal management of inadequate OFS or timing of AI initiation relative to first dose of GnRHa could be studied through simple pragmatic trials.
